# Mediating Role of Parental Support in the Relationship Between Immigrant Mothers’ Mental Health and Adolescents’ Self-Esteem

**DOI:** 10.3390/children12060677

**Published:** 2025-05-24

**Authors:** Yeseul Jeong, Sangyoun Jang

**Affiliations:** 1 College of Nursing, Keimyung University, Daegu 42601, Republic of Korea; yeseuljeong@kmu.ac.kr; 2 Research Institute of Nursing Science, Keimyung University, Daegu 42601, Republic of Korea; 3 Department of Nursing, Catholic Kwandong University, Gangneung 25601, Republic of Korea

**Keywords:** immigrant, parental mental health, parental support, adolescent, self-esteem

## Abstract

Background/Objectives: This study aimed to identify the mediating effect of parental support on the relationship between immigrant mothers’ mental health and adolescents’ self-esteem. Methods: This study utilized data from 1077 Korean multicultural adolescents and their immigrant mothers from the 9th Multicultural Adolescents Panel data obtained in 2019. The data were analyzed using descriptive statistics, Pearson’s correlation, Baron and Kenny’s regression analysis, and bootstrapping using the process macro. Results: Immigrant mothers’ mental health was significantly and positively associated with their adolescents’ self-esteem (r = 0.14, *p* < 0.001), and parental support was also significantly and positively associated with adolescents’ self-esteem (r = 0.50, *p* < 0.001). Parental support had a mediating effect on immigrant mothers’ mental health and adolescents’ self-esteem. Conclusions: The self-esteem of adolescents from multicultural families was found to be influenced by the mental health and support of their immigrant mothers. These findings highlight the mediating role of parental support in the relationship between immigrant mothers’ mental health and adolescents’ self-esteem, contributing to a deeper theoretical understanding of family dynamics in multicultural contexts. Therefore, these factors should be considered when developing parent education programs for immigrant mothers.

## 1. Introduction

The transition from adolescence to adulthood is an important developmental period that requires the achievement of developmental tasks in various areas, such as career planning, forming social relationships, and establishing personal beliefs [1]. In particular, this stage offers freedom and hope to explore various possibilities, leading to a rapid increase in self-esteem. However, it is also marked by heightened anxiety due to uncertainty about the future, which can threaten the formation of a stable self-identity [1,2].

For adolescents approaching the transition to adulthood, self-esteem serves as an internal factor that protects against the psychological stress experienced during this transition process. Major life changes, such as changes in status to adulthood, progression to higher education, entry into the workforce, and expansion of social relationships, demand new roles and responsibilities from adolescents. The pressure caused by these life events can lead to maladaptive outcomes such as anxiety and depression, but self-esteem can act as an important protective factor that prevents these negative effects [3,4]. Since high self-esteem is closely related to an individual’s overall quality of life and long-term psychological health [3,5], it is necessary to identify the factors and pathways that support or hinder its development during this sensitive period as adolescents approach the transition to adulthood.

### 1.1. Challenges to Self-Esteem Development in Immigrant Adolescents: The Korean Context

In immigrant families in Korean society, adolescents face not only significant transitional events in the development of their self-esteem but also additional challenges. Korea has long maintained a homogeneous society, but since the mid-1990s, the sharp increase in international marriages has diversified Korean society ethnically, presenting new challenges for social integration [6,7,8]. In 1990, the proportion of international marriages among all marriages was just 1.2%, but by 2005, it had surged to 13.5% and has remained at 10.1% up to the present [9]. Notably, 80.3% of international marriages involve Korean men and immigrant women [10], a trend driven by women’s educational advancement, expanded social participation, and the resulting marriage difficulties for low-income men. As women’s education levels and social participation have increased, low-income men in rural areas who emphasize traditional values and gender roles have found it more difficult to find spouses domestically, leading to a sharp increase in international marriages facilitated by commercial marriage brokers [6,7]. Amid this increase in international marriages and immigrant populations, immigrant families and their members in Korea have been exposed to social discrimination and prejudice [8,11], which negatively impacts the development of self-esteem in adolescents from immigrant backgrounds [12,13].

In cultures where the mother’s role is crucial in child-rearing, the development of self-esteem in immigrant adolescents is significantly influenced by immigrant mothers. Parental support is essential for the development of children’s self-esteem, but immigrant mothers often face difficulties in providing sufficient support due to cultural differences, language barriers, and social discrimination. For instance, in East Asian cultures influenced by Confucianism, parental, especially maternal, involvement in education is highly valued [14,15]. However, immigrant mothers often face challenges due to differences in values between their home and host countries, language barriers, discrimination, and difficulties in accessing informal information channels such as parent–teacher meetings [16,17,18]. Competence and recognition in areas that adolescents prioritize play a key role in the formation of self-esteem [19]. Therefore, at a time when adolescents are facing important transitions, such as entering higher education and planning their career paths, limited support due to their mothers’ immigration background can lower the self-esteem of teenage children, which can threaten a smooth and healthy transition into adulthood.

### 1.2. Immigrant Mothers’ Mental Health and Adolescents’ Self-Esteem

The psychological difficulties faced by immigrant mothers pose an additional threat to the development of their children’s self-esteem. A previous systematic review [20] has shown that marriage immigrant women in South Korea experience higher rates of overall mental health problems compared to native Korean women and are particularly vulnerable to specific conditions such as depression, postpartum depression, and anxiety. These mental health issues are understood to result from the combined effects of various factors, including socioeconomic conditions (e.g., income and education level), social factors (e.g., marital satisfaction, family relationships, and social support), and cultural factors related to immigration, such as acculturative stress and limited Korean language proficiency [20].

Mental health problems in immigrant parents are not confined to the parents themselves, but are also associated with lower self-esteem in their children [21,22]. The self-esteem, behavioral problems, and depression of youths with immigrant mothers were predicted by the mothers’ sociocultural adaptation, acculturative stress, and perceived discrimination [21]. However, empirical research on the specific pathways linking these issues remains limited.

### 1.3. Potential Mediation by Parental Support

Some studies suggest that the lack of parental support, as perceived by children, may play a crucial mediating role in the relationship between parental mental health problems and the development of children’s self-esteem. Parents with mental health issues tend to have a diminished capacity to provide appropriate support to their children [23], a finding consistently observed in qualitative studies exploring both parent and child experiences. Parents with serious mental health problems often become dependent on their children to meet their needs, failing to respond adequately when their children require support [24]. Additionally, adult children of parents with mental health issues tend to prioritize their parents’ needs over their own, which can lower their self-esteem [25,26,27]. A study by No [28] suggested that the cultural adaptation stress experienced by immigrant mothers reduces parental support and consequently lowers their children’s self-concept. Although this research focused on elementary school children, it suggested that immigrant mothers’ mental health could also impact the self-esteem development of adolescents approaching the transition to adulthood.

### 1.4. In This Study

Previous research has provided fragmented knowledge on the effects of immigrant mothers’ mental health and parental support on the self-esteem development of immigrant adolescents in Korea. Furthermore, empirical studies specifically investigating the pathways through which self-esteem develops in adolescents facing the transition to adulthood, and the protective effects of healthy self-esteem are scarce.

Therefore, this study aims to analyze in detail the impact of immigrant mothers’ mental health, mediated by parental support, on the self-esteem development of adolescents approaching the transition to adulthood. The results of this study could serve as important foundational data for developing policies and interventions aimed at improving the self-esteem of adolescents from immigrant families and supporting their successful transition into adulthood.

## 2. Materials and Methods

### 2.1. Research Design

This study aimed to identify the mediating effect of parental support on the association between immigrant mothers’ mental health and self-esteem among multicultural adolescents and their mothers, using raw data from the 9th year of the Multicultural Adolescent Panel Study in Korea.

### 2.2. Data Source and Study Population

The National Youth Policy Institute collects data from multicultural youths and their parents to improve their school life, social adaptation, physical and psychological health, and parent–child relationships. The Multicultural Adolescents Panel Study, conducted by the National Youth Policy Institute, targets multicultural 4th-grade elementary school students and their mothers in Korea as of 2011. Using stratified random sampling and probability proportional sampling across 16 cities/provinces, the first panel was established in 2011, with data collected annually through the 12th wave in 2023. In this study, the 9th wave of the 1st panel survey of the Multicultural Adolescents Panel Study was used.

The 9th wave of the survey was conducted on 1146 multicultural adolescents and 1114 mothers (1105 foreign mothers). This study included data from immigrant mothers, excluding responses from Korean mothers and cases with missing data. The final sample consisted of 1077 third-year high school students and their immigrant mothers who completed questionnaires regarding key variables: maternal mental health, parental support, and adolescents’ self-esteem.

The process for recruiting panel participants and collecting data was as follows. The National Youth Policy Institute sent an official document to schools with multicultural adolescents requesting their cooperation in recruiting households to participate in the panel survey. The school sent each household a multicultural panel survey-related household newsletter and consent form. Afterwards, the survey was conducted after obtaining voluntary consent from households that wished to participate in the panel survey. This survey was conducted with the approval of the Research Ethics Committee of the National Youth Policy Institute (IRB No. 201904-HR-Goyu-002-01), and this study was approved by the Institutional Review Board of Keimyung University, to which the researcher is affiliated (IRB No. 40525-202502-HR-105-01 and 14 March 2025, of approval). It was conducted by downloading raw data from the National Youth Policy Institute’s data archive after agreeing to the “Data Request Agreement” and “Data Use and Analysis Compliance Guidelines”. The raw data were guaranteed anonymity.

### 2.3. Study Variables

#### 2.3.1. Immigrant Mothers’ Mental Health

The mental health of immigrant mothers was assessed with a modified instrument that Hwang [29] reorganized into 12 items—covering physical and psychological health—from 20 items that Cawte [30] had selected from the Cornell Medical Index Test. A factor analysis conducted on 12 items revealed two factors, ‘physical health’ and ‘mental health’. Most items demonstrated factor loadings of 0.50 or higher, indicating acceptable construct validity. Therefore, all 12 items were retained in the instrument [31]. Six psychological health items were used to assess mental health. The tool uses a 5-point Likert scale. Cronbach’s alpha coefficient was 0.85 in Hwang [29] and 0.88 in our study. In a previous study that used the same instrument with identical participants [32], Cronbach’s alpha was 0.88, the same as in this study.

#### 2.3.2. Parental Support

Parental support was measured using nine items from the Parental Support Tool developed by Kim and Park [33] and extracted from the Multicultural Adolescent Panel Study. The tool consists of emotional, informational, and economic support. Emotional support items include statements such as ‘My parents comfort me when I am having a hard time’, informational support items include ‘My parents give me advice on my future studies’, and financial support items include ‘My parents buy me things I need to study’. The tool was evaluated using a 5-point Likert scale. The scores ranged from 9 to 45, with higher scores indicating higher parental support. Cronbach’s alpha coefficients for each subscale were 0.92 for emotional support, 0.87 for informational support, and 0.82 for economic support. The internal consistency of the overall parental support was 0.93 in Kim and Park [33] and 0.92 in our study. Similarly, a previous study [34], which examined parental support for multicultural adolescents, reported that Cronbach’s alpha was 0.93.

#### 2.3.3. Adolescents’ Self-Esteem

Self-esteem was measured using nine items (five positive and four negative) from the Self-Esteem Scale developed by Rosenberg [35] and extracted from the Multicultural Adolescents Panel Study. It consists of items such as ‘On the whole, I am satisfied with myself’ and ‘I feel that I have a number of good qualities’, ‘I feel that I’m a person of worth’, and ‘I take a positive attitude toward myself’. This tool is measured using a 5-point Likert scale, and negative items are reverse-coded and analyzed. The scores ranged from 9 to 45, with a higher score indicating higher self-esteem. In Rosenberg [35], Cronbach’s alpha ranged from 0.85 to 0.88, whereas it was 0.88 in our study. In a study conducted by Lee and Yoo [36] examining the self-esteem of multicultural adolescents, Cronbach’s alpha coefficient was reported to be 0.88.

### 2.4. Data Analysis

Statistical analyses were performed using SPSS Statistics for Windows, version 29.0 (IBM Corp., New York, NY, USA). The participants’ general characteristics were analyzed by frequency and percentile. Differences in parental support and adolescents’ self-esteem according to general characteristics were analyzed using a *t*-test and analysis of variance. Correlations between immigrant mothers’ mental health, parental support, and adolescents’ self-esteem were analyzed using Pearson’s correlation coefficients.

To assess the mediating effect of parental support on the relationship between immigrant mothers’ mental health and adolescents’ self-esteem, Baron and Kenny’s [37] three-step mediation analysis procedure was employed. The steps were as follows: (1) regression analysis of the mediator on the independent variables, (2) regression analysis of the dependent variable on the independent variables, and (3) regression analysis of the dependent variable on both the independent variables and the mediator. To confirm the statistical significance of the mediating effect, a bootstrapping test was performed using the SPSS Process macro (model 4) with a bootstrap sample size of 1000 and a 95% confidence interval.

## 3. Results

### 3.1. Differences in Parental Support and Adolescents’ Self-Esteem by General Characteristics

The study included 1077 adolescents and their parents. The sex distribution of the adolescents was 49.6% male and 50.4% female. The education levels of mothers and fathers were mostly high school graduates (47.4% and 52.2%, respectively), and 66.7% of the mothers were employed. A significant difference in parental support was observed based on the mother’s education level (*p* = 0.021) and monthly average income (*p* < 0.001). Additionally, adolescents’ self-esteem showed a significant difference based on monthly average income (*p* = 0.036) (Table 1).

### 3.2. The Degree of Immigrant Mothers’ Mental Health, Parental Support, and Adolescents’ Self-Esteem

The mean score for immigrant mothers’ mental health was 4.23 ± 0.73 out of 5 points; the mean score for parental support was 3.93 ± 0.71 out of 5 points, and the mean score for adolescents’ self-esteem was 3.81 ± 0.67 out of 5 points. The normality of the data was assessed using skewness and kurtosis, and all variables were found to follow a normal distribution.

### 3.3. Correlations Among Immigrant Mothers’ Mental Health, Parental Support, and Adolescents’ Self-Esteem

The mental health of immigrant mothers showed a significant positive correlation with parental support (r = 0.11, *p* < 0.001) and adolescents’ self-esteem (r = 0.14, *p* < 0.001). Parental support had a significant positive correlation with adolescents’ self-esteem (r = 0.50, *p* < 0.001) (Table 2).

### 3.4. Mediating Effect of Parental Support in the Relationships Between Immigrant Mothers’ Mental Health and Adolescents’ Self-Esteem

To verify the mediating effect of parental support on the relationship between immigrant mothers’ mental health and adolescents’ self-esteem, we performed the three-step mediation effect verification procedure outlined by Baron and Kenny [37] and conducted the Process macro to assess the statistical significance of the mediation effect.

During the regression analysis, we checked for multicollinearity. The tolerance limit was 0.95 to 0.96, which was above the threshold of 0.1, and the variance inflation factor (VIF) was 1.014, well below the cutoff of 10, indicating that multicollinearity was not a concern. In the test for the autocorrelation of errors, the Durbin–Watson index was 1.952, which is close to 2, indicating that there was no autocorrelation. After verifying the assumptions of normality and homoscedasticity of the residuals, the normal P-P plot of the standardized residuals satisfied normality. The residuals were evenly distributed above and below zero in the scatter plot, satisfying the equal variance of the residuals. Therefore, all the basic assumptions of the regression analysis were satisfied.

In the mediation effect analysis, to more clearly confirm the mediating effect of parental support on the immigrant mothers’ mental health and the adolescents’ self-esteem, the mothers’ education level and monthly household income—identified as statistically significant variables in the univariate analyses—were included as control variables in the mediation model.

**Figure 1:**
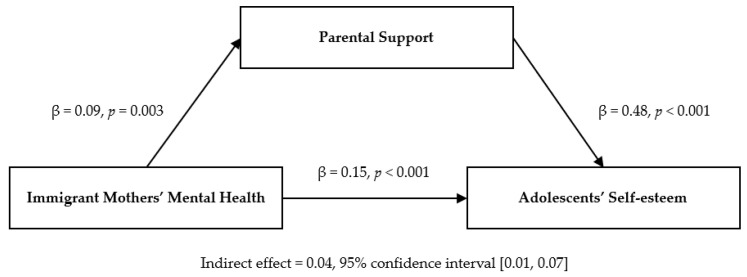
Mediating effect of parental support in the association between immigrant mothers’ mental health and adolescents’ self-esteem.

Table 3 presents the results of the three-step mediation effect verification procedure based on Baron and Kenny’s [37] model. In the first step, the effect of the independent variable, immigrant mothers’ mental health, on the mediator, parental support, was statistically significant (β = 0.09, *p* = 0.003). In the second step, the independent variable, immigrant mothers’ mental health, on the dependent variable, adolescents’ self-esteem, was also statistically significant (β = 0.15. *p* < 0.001). In the third step, parental support significantly affected the adolescents’ self-esteem (β = 0.48, *p* < 0.001). Notably, the β value for the effect of immigrant mothers’ mental health on adolescents’ self-esteem in the third step was 0.11 (*p* < 0.001), which was lower than the β value in the second step (β = 0.15, *p* < 0.001), indicating that parental support partially mediated the relationship between immigrant mothers’ mental health and adolescents’ self-esteem (Figure 1).

The significance of the indirect mediation effect was tested using bootstrapping analysis. The effect size was 0.04, which was found to be statistically significant because zero was not included between the 95% lower limit confidence interval (BootLLCI = 0.01) and the upper limit confidence interval (BootULCI = 0.07).

## 4. Discussion

This study examined the level of immigrant mothers’ mental health, parental support, and adolescents’ self-esteem and investigated whether parental support mediates the relationship between immigrant mothers’ mental health and adolescents’ self-esteem.

### 4.1. Influrence of Socioeconomic Factors on Parental Support and Adolescents’ Self-Esteem

Significant differences in parental support and adolescents’ self-esteem were observed based on their general characteristics. Parental support differed significantly depending on the mother’s education level and the household’s average monthly income. This study found that parental support was higher among mothers who had completed a four-year college degree or had higher income levels. This finding aligns with previous studies indicating that parents with higher educational attainment tend to provide better emotional support than those with lower education levels [38].

Also, adolescents’ self-esteem showed significant differences by average monthly household income. In this study, higher income levels were associated with higher self-esteem among adolescents. These results are consistent with meta-analysis studies showing that parents with higher household income have more positive parenting behaviors [39], as well as prior research showing that children from families with higher socioeconomic status tend to exhibit greater self-esteem [40]. These results suggest that adolescents from higher-income families may benefit from greater financial stability and access to resources, which in turn contribute to enhanced self-esteem.

In contrast, parents with lower educational and socioeconomic status often face constraints due to long working hours, limited time for family interaction, and challenges in fulfilling supportive roles for their children [38]. In light of these results, immigrant mothers should be offered regular educational programs and counseling to improve their communication skills and ability to provide positive support for their children. Furthermore, it is essential to prioritize socioeconomic support for multicultural families with low levels of education and income.

### 4.2. Relationships Among Immigrant Mothers’ Mental Health, Parental Support, and Adolescents’ Self-Esteem in Multicultural Families

This study found significant positive correlations among immigrant mothers’ mental health, parental support, and adolescents’ self-esteem. Lee and Yoo [36] similarly found that poorer physical and psychological health among married immigrant women was associated with lower adolescent self-esteem, supporting the present finding that better maternal mental health corresponds with higher adolescent self-esteem. However, since previous studies have often treated the physical and mental health of immigrant mothers as a single variable, there is a limitation in making direct comparisons with the mental health of this study. Nevertheless, the current finding is meaningful in that it confirms the influence of mothers on adolescents’ self-esteem. In addition, Um’s study [41], which focused on multicultural adolescents, reported that parental support was significantly positively correlated with adolescents’ self-esteem. Um [41] found that parental support significantly influenced self-esteem (β = 0.06, *p* < 0.001), consistent with the present study’s finding that parental support has a significant impact on self-esteem (β = 0.48, *p* < 0.001).These results demonstrate the importance of mental health and support for immigrant mothers.

In this way, significant correlations were identified among the major variables. However, the association between immigrant mothers’ mental health and adolescents’ self-esteem was relatively small. One possible explanation is that in South Korea, the majority of multicultural families have immigrant mothers who often experience emotional difficulties such as depression and anxiety during the process of cultural adaptation, communication problems, and interpersonal relationship difficulties in the host country [20]. Given that mothers in Korean families typically play a more important role in child-raising and education, immigrant mothers may experience increased stress and burden when their parenting values and practices differ from those of their home country.

The cultural adaptation stress of immigrant mothers was found to indirectly affect the development of children’s self-concept through parenting efficacy and parental support [28]. The findings suggest that mothers’ cultural adaptation stress reduces their parenting efficacy, lowering their parents’ support level and ultimately hindering the development of self-concept in adolescent children. In addition, parenting stress has been reported to be higher in vulnerable groups with lower education and income levels [42]. A systematic literature review examining the relationship between household income and child development outcomes found that low socioeconomic status negatively impacts children’s cognitive and socio-behavioral development and health [43]. In line with these findings, a meta-analysis study conducted by Ayoub and Bachir [39] reported that mothers from high-income families showed more positive parenting behaviors than those from low-income families and that parental supportive behavior had an effect on increasing children’s self-esteem [21].

According to Statistics Korea [44], the standard median income in Korea is approximately KRW 5 million or more for a three-person household and approximately KRW 6 million or more for a four-person household. In this study, although most parents were employed (86.0% of fathers, 66.7% of mothers), 77.3% of households reported an average monthly household income below KRW 4 million. This indicates that most multicultural families in this study were of low socioeconomic status, showing a notable income gap compared to the general population.

Given these findings, the dataset primarily reflects low-income families, which may have limited the variability necessary to detect stronger associations between immigrant mothers’ mental health and adolescents’ self-esteem. Meanwhile, this study is meaningful in that it identified a strong correlation between parental support and self-esteem and confirmed the mediating effect of parental support between immigrant mothers’ mental health and self-esteem. Self-esteem formed through a supportive relationship between parents and their children in early adolescence influences mental well-being in later adolescence [45]. Therefore, further research is needed to confirm the influence of parental support in the future.

### 4.3. Parental Support as a Mediator Between Immigrant Mothers’ Mental Health and Adolescents’ Self-Esteem

This study identified a significant mediating effect of parental support on the relationship between immigrant mothers’ mental health and adolescents’ self-esteem. It was difficult to find previous studies that confirmed the mediating effect of parental support on the same variables as in this study; however, we can discuss them in connection with studies that confirmed the relationship between each variable. A systematic literature review of the relationship between the mental health of immigrant parents and multicultural adolescents in other countries reported that when immigrant parents have mental stress, anxiety, and depression symptoms, it has a negative impact on their children’s stress and anxiety symptoms, as well as their self-esteem [21]. In addition, it is generally known that adolescents’ self-esteem is affected by their relationship with their parents, and positive interactions with parents, parental support, and encouragement have a significant effect on adolescents’ self-esteem [41,46]. Moreover, when parents have a warm and supportive parenting style, their children have been shown to have high self-esteem [21,47]. This finding suggests the need to strengthen parental support systems for multicultural adolescents to achieve stable self-esteem. Therefore, to improve adolescents’ self-esteem, a plan to improve mothers’ mental health so that they can positively support their children is necessary.

### 4.4. Limitations and Implications

The limitations of this study are as follows. First, this study has methodological limitations because it analyzed cross-sectional data from 18-year-old multicultural adolescents and their immigrant mothers. Future studies need to verify the longitudinal mediating effect of parental support on adolescents’ self-esteem. Second, the mental health of immigrant mothers was assessed through a self-report questionnaire, which may be prone to response bias due to subjective perception. The use of physiological indicators could improve the objectivity of mental health assessments. Third, this study focused on the effects of immigrant mothers’ mental health and support on adolescents’ self-esteem. Future research will also be needed to analyze the effects of Korean fathers’ mental health and support on adolescents’ self-esteem. Additionally, further studies should incorporate other potentially influential factors such as peer relationships and teacher support to provide a more comprehensive understanding of the determinants of self-esteem in multicultural adolescents. Fourth, as this study was conducted with multicultural families residing in Korea, its findings may be influenced by cultural and contextual factors unique to the Korean setting, which may limit generalizability to other countries. Lastly, this study has a limitation in that it did not control for factors such as social discrimination or stigma arising from the mother’s mental health condition, which could potentially mediate the relationship between maternal mental health and adolescents’ self-esteem. Accordingly, future research should explicitly incorporate this variable to clarify its influence. Nevertheless, this study has the strength of generalizing the results by analyzing representative large-scale data from the Multicultural Adolescent Panel Study. Additionally, this study confirmed the mediating effect of parental support on immigrant mothers’ mental health and adolescents’ self-esteem.

The theoretical and practical implications of this study are as follows. From a theoretical perspective, this study contributes to theoretical models of adolescent development by empirically demonstrating the mediating role of parental support in the relationship between immigrant mothers’ mental health and adolescents’ self-esteem. This study extends prior research by contextualizing these dynamics within multicultural families.

With regard to practical implications, the findings suggest that programs aiming to enhance adolescents’ self-esteem in multicultural families should not only focus on adolescent-centered interventions but also prioritize the mental health of immigrant mothers and strengthen their parenting capacities. Policy makers and practitioners should develop culturally sensitive parent education and mental health support services that empower immigrant mothers in their parenting roles.

## 5. Conclusions

This study was conducted to identify the mediating effect of parental support on the relationship between immigrant mothers’ mental health and adolescents’ self-esteem, with the goal of providing foundational data for developing nursing interventions to enhance adolescents’ self-esteem.

The findings confirmed that immigrant mothers’ mental health significantly influenced their adolescents’ self-esteem. Furthermore, parental support was found to partially mediate the relationship between the mothers’ mental health and the adolescents’ self-esteem. Therefore, to improve adolescents’ self-esteem in multicultural families, intervention strategies targeting both parental support and immigrant mothers’ mental health are essential.

Additionally, the study identified that parental support had a significant impact on adolescents’ self-esteem, with variations depending on their level of education and household income. Based on these findings, this study suggests the development of an educational program aimed at mothers with lower education and income levels to support their children both emotionally and educationally. 

## Figures and Tables

**Table 1:** General characteristics of the participants (*N* = 1077).

Variable	Category	n (%)	Parental Support (*n* = 1077)			Adolescents’ Self-Esteem (*n* = 1077)		
			Mean ± SD	t/F	*p*	Mean ± SD	t/F	*p*
Sex	Male adolescent	534 (49.6)	35.56 ± 6.56	0.98	0.326	34.62 ± 5.89	1.75	0.080
	Female adolescent	543 (50.4)	35.18 ± 6.18			33.98 ± 6.13		
Mother’s education	Middle school or below ^a^	114 (10.6)	34.04 ± 6.77	2.90	0.021 a < e ^†^	33.26 ± 6.62	2.32	0.055
	High school ^b^	510 (47.4)	35.65 ± 6.17			34.19 ± 6.04		
	College ^c^	284 (26.4)	35.13 ± 6.64			34.51 ± 5.87		
	University ^d^	164 (15.2)	35.65 ± 6.10			34.80 ± 5.63		
	Graduate school ^e^	5 (0.5)	41.60 ± 4.51			39.80 ± 6.72		
Father’s education *	Middle school or below	335 (32.4)	35.05 ± 6.55	0.74	0.563	34.43 ± 6.07	0.39	0.819
	High school	540 (52.2)	35.50 ± 6.15			34.29 ± 6.03		
	College	59 (5.7)	35.92 ± 6.91			34.64 ± 5.37		
	University	91 (8.8)	36.22 ± 6.54			34.81 ± 6.21		
	Graduate school	9 (0.9)	35.44 ± 6.75			36.22 ± 5.09		
Mother’s occupation	Yes	718 (66.7)	35.46 ± 6.26	−0.68	0.499	34.46 ± 5.92	−1.23	0.219
	No	359 (33.3)	35.18 ± 6.59			33.98 ± 6.21		
Father’s occupation *	Yes	888 (86.0)	35.50 ± 6.44	−0.73	0.469	34.50 ± 6.02	−1.25	0.211
	No	145 (14.0)	35.08 ± 6.09			33.83 ± 5.89		
Monthly household income (KRW 10,000) *	<100 ^a^	16 (1.5)	35.38 ± 6.52	4.71	<0.001 a < f ^‡^	34.94 ± 5.11	2.40	0.036 b < f ^‡^
	100~< 200 ^b^	198 (18.4)	33.82 ± 6.99			33.49 ± 6.63		
	200~<300 ^c^	308 (28.6)	35.18 ± 6.26			34.06 ± 5.73		
	300~<400 ^d^	310 (28.8)	35.55 ± 6.28			34.20 ± 5.90		
	400~<500 ^e^	152 (14.1)	36.22 ± 6.02			35.02 ± 6.08		
	≥500 ^f^	92 (8.6)	37.27 ± 5.42			35.75 ± 5.70		

* Excludes non-responses. ^†^ Tukey HSD post hoc test; the mean difference is significant at the 0.10 level. ^‡^ Tukey HSD post hoc test; the mean difference is significant at the 0.05 level. The footnotes in a–f were used to indicate the results of the post-analysis.

**Table 2:** Correlations among the variables (*N* = 1077).

Variable	Immigrant Mothers’ Mental Health	Parental Support
Parental support	0.11 (<0.001)	
Adolescent self-esteem	0.14 (<0.001)	0.50 (<0.001)

**Table 3:** Mediating effect of parental support on the relationship between immigrant mothers’ mental health and adolescents’ self-esteem (*N* = 1077).

Variable	B	SE	β	t	*p*	95% CI	R^2^	Adj.R^2^	F	*p*
Step 1. Immigrant mother’s mental health → parental support	0.13	0.04	0.09	3.02	0.003	[0.05, 0.22]	0.040	0.032	4.64	<0.001
Step 2. Immigrant mother’s mental health → adolescent self-esteem	0.21	0.04	0.15	5.02	<0.001	[0.13, 0.29]	0.041	0.032	4.65	<0.001
Step 3. Immigrant mother’s mental health, parental support → adolescent self-esteem							0.260	0.253	35.16	<0.001
Immigrant mother’ mental health → adolescent self-esteem	0.15	0.04	0.11	4.06	<0.001	[0.08, 0.22]				
Parental support → adolescent self-esteem	0.45	0.03	0.48	18.07	<0.001	[0.40, 0.50]				

Note. CI = confidence interval.

## Data Availability

The data presented in this study are openly available at the National Youth Policy Institute (https://www.nypi.re.kr/archive, accessed on 4 February 2025).
